# Association between triglyceride-glucose index and hypertension: a cohort study based on the China Health and Nutrition Survey (2009–2015)

**DOI:** 10.1186/s12872-024-03747-9

**Published:** 2024-03-19

**Authors:** Su Wang, Qian Wang, Xianliang Yan

**Affiliations:** Department of Cardiology, Beijing Anzhen Hospital, Capital Medical University, Beijing, 100029 China

**Keywords:** Hypertension, TyG index, CHNS, Retrospective cohort study

## Abstract

**Aim:**

Insulin resistance (IR) may participate in the pathogenesis of hypertension by mediating low-grade systemic inflammation. The triglycerides-glucose (TyG) index has recently been suggested as a reliable alternative biochemical marker of IR compared with traditional methods. Herein, we speculated TyG index may also be associated with hypertension.

**Methods:**

Data of adults were extracted from the China Health and Nutrition Survey (CHNS) in 2009–2015 in this retrospective cohort study. The TyG index was calculated using the formula: TyG = Ln [fasting triglycerides (mg/dL) ×fasting glucose (mg/dL)/2]. Associations between TyG index and hypertension were evaluated by univariate and multivariate logistic regression analyses with odds ratios (ORs) and 95% confidence intervals (CIs). Subgroup analyses of age and gender were also performed. In addition, we assessed the interaction effect between TyG index and body mass index (BMI) on hypertension in participants with different age and gender.

**Results:**

Among 3,413 eligible participants, 1,627 (47.67%) developed hypertension. The average TyG index in hypertension group and non-hypertension group was 8.58 and 8.39 respectively. After adjusting for covariates, we found that compared with participants with TyG index ≤ 8.41 (median value), those who had higher TyG index seemed to have higher odds of hypertension [OR = 1.17, 95%CI: (1.01–1.37)]. Similarly, this association was also discovered in participants who aged ≤ 65 years old [OR = 1.19, 95%CI: (1.01–1.39)] or were female [OR = 1.35, 95%CI: (1.10–1.65)]. Additionally, there was a potential additive interaction effect between obesity and TyG index on hypertension.

**Conclusion:**

High TyG index was associated with high odds of hypertension in general population in China, but the causal relationship between them needed further exploration.

**Supplementary Information:**

The online version contains supplementary material available at 10.1186/s12872-024-03747-9.

## Introduction

Hypertension is one of the most prevalent chronic medical diseases of adults, and has been reported to be the most important modifiable risk factor for cardiovascular disease (CVD) [1]. Hypertension affects about 1 billion adults and is associated with more than 9 million deaths annually in the United States [2]. In China, hypertension has also posed a major public health challenge [3]. A nationally represented survey in 2012–2015 found that almost half the hypertensive population was aware of their hypertension, 40.7% were treated, and however, only 15.3% achieved blood pressure control [4]. Therefore, understanding how to prevent hypertension and early identify hypertensive patients are critical to reduce cardiovascular mortality and lower the burden of both disease and socioeconomic.

Excess adiposity may be a major culprit associated with the causes of primary hypertension, which are although not completely clarified. As much as 65–75% of the risk for primary hypertension can be attributed to excess weight gain and obesity in multiple populations [5]. Epidemiological studies have also documented a high incidence of diabetes mellitus (DM) in hypertensive patients [6, 7]. Due to the insulin resistance (IR) plays a pivotal role in the pathogenesis of type 2 DM, it may also participate in the pathogenesis of hypertension [7, 8]. IR can mediate low-grade systemic inflammation, which is closely related to hypertension and is an important pathological mechanism of elevated blood pressure [9]. Therefore, early identification of IR in general population may have important clinical significance for the management of hypertension.

The most mature method of IR measurement so far is the hyper insulinemic-euglycemic clamp test, which is usually considered as the “gold standard” method for insulin sensitivity assessment [10, 11]. However, due to the high cost of time and money, the extensive applicability of this method is limited. Another kind of method to measure IR is using the steady-state model assessment (HOMA) of IR, which is an indirect method that requires insulin measurement, so that it is often difficult to repeat the same results again [12, 13]. Besides, the triglycerides-glucose (TyG) index is an index that not based on insulin, which is calculated by the fasting triglycerides and logarithmic product of fasting plasma glucose [14, 15]. The TyG index is associated with lipotoxicity and glucotoxicity (which is key factors in the IR adjustment), and has been suggested as a reliable alternative biochemical marker of IR [12]. Moreover, TyG index is low-priced and can be easily accessed from a single sample.

Herein, our study adopted a cohort study design and aimed to explore the association between TyG index and hypertension in Chinese population, in order to provide some references for the identification and management of high-risk population of hypertension early, and in addition, to employ simpler and cheaper measures for early screening of hypertension.

## Methods

### Study design and data source

This is a retrospective cohort study based on the China Health and Nutrition Survey (CHNS) database in 2009–2015. The CHNS is a subsequent follow-up survey of nutrition and food safety of the Chinese Center for Disease Control and Prevention (CCDC) in collaboration with the Population Center of the University of North Carolina (UNC) in the United States. CHNS covered nine provinces (Liaoning, Heilongjiang, Jiangsu, Shandong, Henan, Hubei, Hunan, Guangxi, and Guizhou), and used multistage and random cluster process to draw the samples surveyed in each of the provinces. Four counties were randomly selected after stratified by income (low, middle, and high) in each province. In addition, selecting a provincial capital and a lower income city when feasible, except in two provinces where large cities rather than provincial capitals had to be selected. Villages and townships within the counties and urban/suburban neighborhoods within the cities were selected randomly. Details of the CHNS survey data have been described elsewhere [16].

After the multistage and random cluster process, subjects of the CHNS are representative Chinese population. Apparently healthy participants attended the baseline visit, during which they gave their informed consent and completed a structured questionnaire that asked about socioeconomic characteristics, lifestyle exposures, general health, and medical history. No ethical approval for this study was required from the Institutional Review Board (IRB) of Beijing Anzhen Hospital, Capital Medical University, because the data was accessed from CHNS (a publicly available database). All the study methods were carried out in accordance with relevant guidelines and regulations (declaration of Helsinki).

### Participants

Data of 6,874 adults without hypertension were extracted from the CHNES in 2009–2015. The exclusion criteria were (1) age < 18 years old, and (2) without the information of triglyceride (TG) and fasting blood glucose (FBG) which used to calculate TyG. Also, those who missing the information of smoking, asthma, body mass index (BMI), DM, height, weight, and low-density lipoprotein (LDL) were excluded. Finally, 3,413 participants were eligible.

### Diagnosis of hypertension

According to a standard protocol, BP was measured by trained examiners using a mercury sphygmomanometer with a suitable cuff size. Triplicate measurements were taken 10 min after rest, and the documented individual’s BP was the average of the three measurements. BP ≥ 130/80 mmHg or use of antihypertensive medications within the previous 2 weeks was diagnosed as hypertension according to the 2017 the American College of Cardiology and American Heart Association (ACC/AHA) guideline. Identical to 2018 Chinese hypertension guideline, stage 1 hypertension was defined by systolic blood pressure (SBP) of 130–139 mm Hg or diastolic blood pressure (DBP) of 80–89 mm Hg, and stage 2 hypertension was defined as SBP ≥ 140 or DBP ≥ 90 mmHg or the use of antihypertensive medications [17, 18]. Therefore, the diagnosis criteria of adult hypertension were SBP ≥ 130 mmHg or DBP ≥ 80 mmHg or the use of antihypertensive medications.

### Calculation of TyG index

TyG index was calculated using the formula: TyG = Ln [fasting triglycerides (mg/dL) ×fasting glucose (mg/dL)/2]. Herein, we extracted the information of baseline fasting triglycerides and fasting glucose of the participants from the CHNS database to calculate the TyG index [19].

### Outcome and follow-up

The study outcome was the occurrence of hypertension. The time end-point of the follow-up were respectively at 31st December 2009, 31st December 2011, and 31st December 2015.

### Variables collection

We also collected variables including age, gender, education level, weight, height, BMI [calculated by the ratio of weight (kg) to the square of height (m)], marital status, smoking, drinking, DM, asthma (identified using ICD-9 codes), total cholesterol (TC), high density lipoprotein (HDL), LDL, insulin injection use, fasting glucose, and triglyceride.

### Statistical analysis

Mean and standard deviation (mean ± SD) was used to describe the distribution of normally distributed data, and t test was used to compare the difference between two groups. Median and quartiles [M (Q1, Q3)] were used to describe the abnormally distributed data, and Mann-Whitney U rank sum test was used for the comparation. The frequency and composition ratio [N (%)] was used to describe the distribution of categorical data, and chi-square test (χ2) was used for the comparation.

Univariate logistic regression analysis was used for covariates screening. Univariate and multivariate logistic regression analyses were used to explore the association between TyG index and hypertension. Model 1 was the crude model. Model 2 adjusted for age, gender, education level, BMI (we only taken BMI into adjustment to avoid the multicollinearity), and marital status. Model 3 additionally adjusted smoking, drinking, DM, TC, and LDL based on Model 2. The restricted cubic spline (RCS) curve was plotted to determine whether the TyG index had nonlinear trends. Subgroup analysis of age and gender were also performed. In addition, we assessed the potential interaction effect between TyG index and obesity on hypertension among total population and subgroup populations respectively. The evaluated index was odds ratios (ORs) and confidence intervals (CIs). Bilateral *P* < 0.05 was considered have significant difference.

Statistics analyses were completed by SAS 9.4 (SAS Institute., Cary, NC, USA) and R software v 4.0.3 (urt 2020-10-10). Missing variables were showed in Table S1, and we performed the sensitivity analysis of characteristics of participants before and after the deletion of missing variables (Table S2).

## Results

### Characteristics of participants

**Fig. 1:**
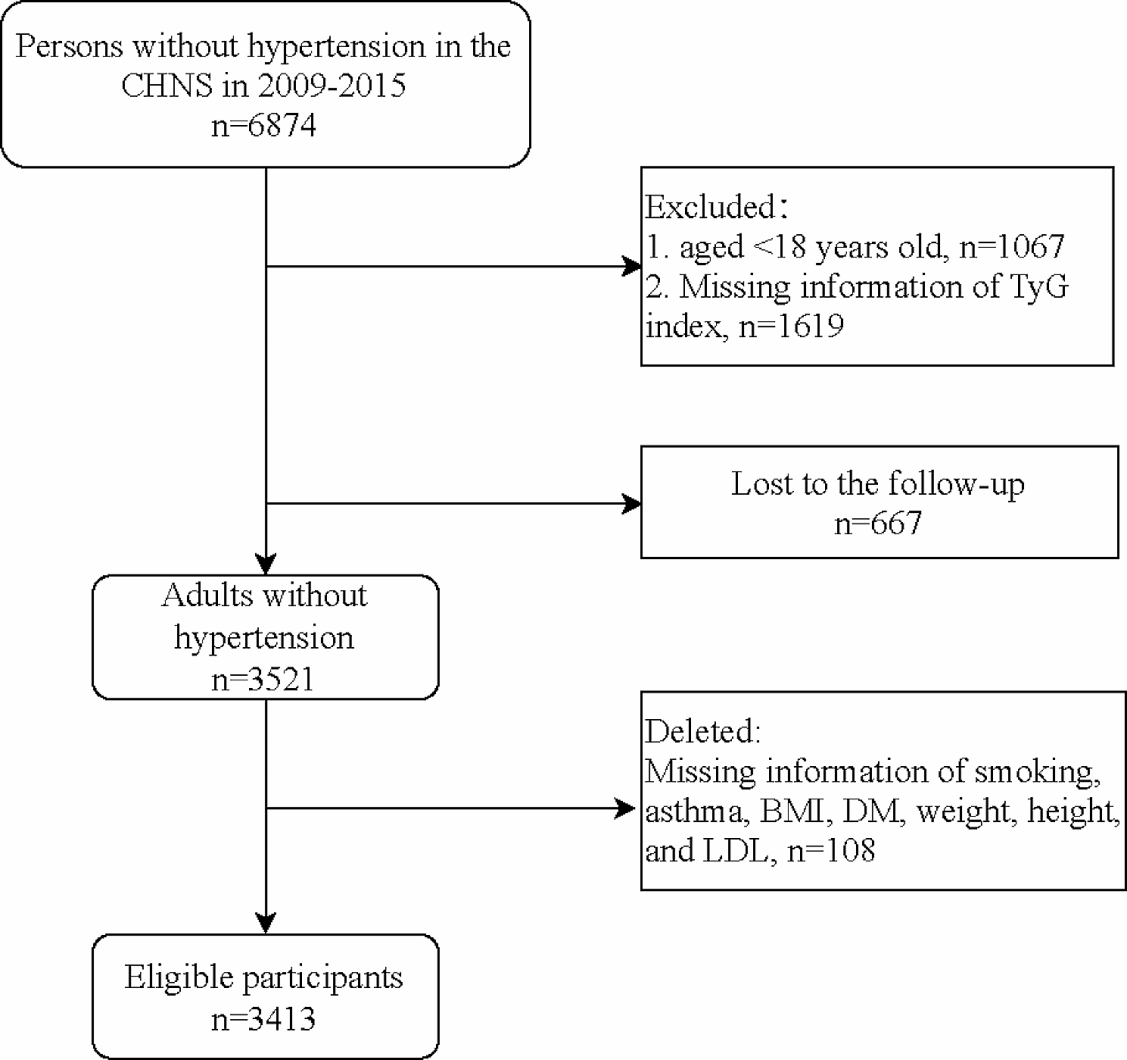
Flowchart of participants screening

Figure 1 showed the flowchart of participants screening. We initially included 6,874 individuals without hypertension from the CHNS database. Then, those who aged < 18 years old (*n* = 1067) or missing information of TyG index (*n* = 1619) were excluded. During 2009–2015, 667 of them lost to the follow-up. Also, we excluded participants who missing the information of smoking, asthma, BMI, DM, weight, height, and LDL (*n* = 108). Finally, 3,413 adults were eligible.

Table 1 showed the characteristics of the persons in non-hypertension group and hypertension group. The average age of eligible participants was 46.24 years old, and 1,998 (58.54%) were female. The average BMI between non-hypertension group and hypertension group was 21.88 kg/m^2^ and 23.16 kg/m^2^. The TyG index between these two groups was respectively 8.39 and 8.58. Moreover, age, gender, education level, marital status, smoking, drinking, DM, weight, BMI, TC, LDL, fasting glucose, and triglyceride were all significantly different between non-hypertensive persons and hypertensive persons (all *P* < 0.05).

**Table 1:** Characteristics of eligible participants

Variables	Total (*n* = 3413)	Non-hypertension (*n* = 1786)	Hypertension (*n* = 1627)	Statistics	*P*
Age, years, Mean ± SD	46.24 ± 13.92	43.30 ± 14.05	49.46 ± 13.03	t=-13.27	< 0.001
Gender, n (%)				χ2 = 22.681	< 0.001
Male	1415 (41.46)	672 (37.63)	743 (45.67)		
Female	1998 (58.54)	1114 (62.37)	884 (54.33)		
Education level, n (%)				χ2 = 20.939	< 0.001
Lower middle school degree	2542 (74.48)	1272 (71.22)	1270 (78.06)		
Upper middle school degree	871 (25.52)	514 (28.78)	357 (21.94)		
Marital status, n (%)				χ2 = 79.144	< 0.001
Married	1213 (35.54)	759 (42.50)	454 (27.90)		
Others (divorced, separated, single, unknown)	2200 (64.46)	1027 (57.50)	1173 (72.10)		
Smoking, n (%)				χ2 = 20.120	< 0.001
No	2456 (71.96)	1344 (75.25)	1112 (68.35)		
Yes	957 (28.04)	442 (24.75)	515 (31.65)		
Drinking, n (%)				χ^2^ = 16.908	< 0.001
No	1040 (30.47)	489 (27.38)	551 (33.87)		
Yes	2373 (69.53)	1297 (72.62)	1076 (66.13)		
DM, n (%)				χ2 = 7.351	0.007
No	3374 (98.86)	1774 (99.33)	1600 (98.34)		
Yes	39 (1.14)	12 (0.67)	27 (1.66)		
Asthma, n (%)				χ2 = 0.385	0.535
No	3381 (99.06)	1771 (99.16)	1610 (98.96)		
Yes	32 (0.94)	15 (0.84)	17 (1.04)		
Weight, kg, Mean ± SD	58.03 ± 10.09	56.25 ± 9.42	59.98 ± 10.44	t=-10.94	< 0.001
Height, m, Mean ± SD	160.39 ± 8.25	160.13 ± 8.21	160.68 ± 8.29	t=-1.94	0.053
BMI, kg/m^2^, Mean ± SD	22.49 ± 3.10	21.88 ± 2.93	23.16 ± 3.15	t=-12.23	< 0.001
TC, mg/dL, Mean ± SD	182.50 ± 37.40	177.67 ± 37.16	187.79 ± 36.96	t=-7.97	< 0.001
HDL, mg, Mean ± SD	56.51 ± 17.45	56.87 ± 16.75	56.11 ± 18.19	t = 1.27	0.203
LDL, mg, M (Q_1_, Q_3_)	111.17 ± 36.08	107.43 ± 35.26	115.27 ± 36.54	t=-6.37	< 0.001
Insulin injection use, M (Q_1_, Q_3_)	0.00 (0.00, 1.00)	0.00 (0.00, 0.00)	0.00 (0.00, 1.00)	Z=-1.306	0.191
Fasting glucose, mg/dL, Mean ± SD	92.83 ± 21.99	90.96 ± 21.04	94.89 ± 22.83	t=-5.22	< 0.001
Triglyceride, mg/dL, M (Q_1_, Q_3_)	99.20 (69.97, 151.46)	93.00 (65.54, 140.83)	108.06 (76.17, 163.86)	Z = 7.555	< 0.001
TyG index, Mean ± SD	8.48 ± 0.65	8.39 ± 0.64	8.58 ± 0.65	t=-8.64	< 0.001
TyG index level, n (%)				χ2 = 59.713	< 0.001
≤8.41	1707 (50.01)	1006 (56.33)	701 (43.09)		
>8.41	1706 (49.99)	780 (43.67)	926 (56.91)		

SD: standard difference, DM: diabetes mellitus, TC: total cholesterol, M: median, BMI: body mass index, HDL: high density lipoprotein, LDL: low density lipoprotein, TyG index: triglycerides-glucose index

t: t-test, Z: rank sum test, χ2: chi-square test

**Fig. 2:**
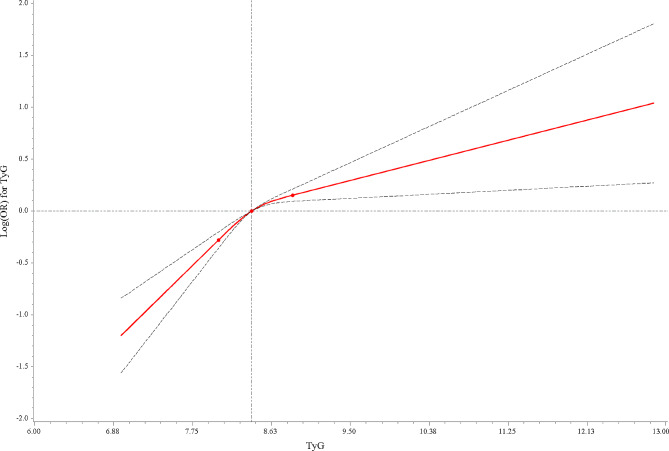
Restricted cubic spline (RCS) curve of the TyG index

In addition, Fig. 2 was the RCS curve of TyG index. The results showed that TyG index has a nonlinear trend, indicating that it may be more meaningful to be served as TyG a categorical variable in further analyses. So that we used both continuous TyG index and categorical TyG index (which was classified into two levels according to the median value 8.41) to explore the association between it and hypertension.

### Association between TyG index and hypertension

According to the Table 1, we included the variables significantly different between non-hypertension group and hypertension group into adjustment in multivariate models. Then we explored the association between TyG index and hypertension (Table 2). After adjusting for the covariates, there was no significantly association between the increased TyG index and the odds of hypertension (*P* = 0.92). However, compared with adults who had TyG index ≤ 8.41, those who had TyG index > 8.41 seemed to have higher odds of hypertension [OR = 1.17, 95%CI: (1.01–1.37)].

**Table 2:** Association between TyG index and hypertension

Variables	Model 1		Model 2		Model 3	
	OR (95% CI)	*P*	OR (95% CI)	*P*	OR (95% CI)	*P*
TyG index	1.59 (1.43–1.77)	< 0.001	1.19 (1.06–1.34)	0.004	1.13 (0.99–1.29)	0.080
TyG index level						
≤8.41	Ref		Ref		Ref	
>8.41	1.70 (1.49–1.95)	< 0.001	1.24 (1.07–1.44)	0.004	1.17 (1.01–1.37)	0.047

TyG index: triglycerides-glucose index, OR: odds ratio, CI: confidence interval, Ref: reference

Model 1: crude model;

Model 2: adjusted for age, gender, education level, BMI, marital status;

Model 3: adjusted for age, gender, education level, BMI, marital status, smoking, drinking, DM, asthma, TC, and LDL.

### Relationship between TyG index and hypertension in subgroups of age and gender

We further explored the relationship between TyG index and hypertension among adults with different age (the cut-off value was 65 years old) and gender (Table 3). After adjusting for covariates, we only found the odds of hypertension increased along with every additional unit of TyG index in females [OR = 1.20, 95%CI: (1.01–1.45)]. In participants who aged < 65 years old [OR = 1.19, 95%CI: (1.01–1.39)] or were female [OR = 1.35, 95%CI: (1.10–1.65)], higher TyG index was associated with higher odds of hypertension compared with TyG index ≤ 8.41.

**Table 3:** Association between TyG index and hypertension in subgroups of age and gender

Variables	Model 1		Model 2		Model 3	
	OR (95% CI)	*P*	OR (95% CI)	*P*	OR (95% CI)	*P*
**< 65 years old (*n*** = **3076)**						
TyG index	1.60 (1.43–1.79)	< 0.001	1.20 (1.07–1.36)	0.003	1.15 (1.01–1.32)	0.062
TyG index level						
≤8.41	Ref		Ref		Ref	
>8.41	1.73 (1.50–1.99)	< 0.001	1.24 (1.06–1.45)	0.007	1.19 (1.01–1.39)	0.040
**≥ 65 years old** (***n*** = **337)**						
TyG index	1.30 (0.88–1.92)	0.194	1.01 (0.64–1.59)	0.965	1.06 (0.65–1.72)	0.825
TyG index level						
≤8.41	Ref		Ref		Ref	
>8.41	1.46 (0.94–2.27)	0.094	1.20 (0.74–1.96)	0.464	1.20 (0.73-2.00)	0.473
**Male** (***n*** = **1415)**						
TyG index	1.38 (1.19–1.61)	< 0.001	1.13 (0.96–1.34)	0.148	1.01 (0.82–1.24)	0.924
TyG index level						
≤8.41	Ref		Ref		Ref	
>8.41	1.36 (1.10–1.68)	0.004	1.04 (0.83–1.31)	0.728	0.92 (0.72–1.18)	0.514
**Female** (***n*** = **1998)**						
TyG index	1.75 (1.50–2.03)	< 0.001	1.22 (1.04–1.45)	0.018	1.20 (1.01–1.45)	0.055
TyG index level						
≤8.41	Ref		Ref		Ref	
>8.41	1.96 (1.64–2.34)	< 0.001	1.38 (1.13–1.67)	0.001	1.35 (1.10–1.65)	0.004

TyG index: triglycerides-glucose index, OR: odds ratio, CI: confidence interval, Ref: reference

Model 1: crude model;

Model 2: adjusted for age (not included in age subgroup), gender (not included in gender subgroup), education level, BMI, marital status;

Model 3: adjusted for age (not included in age subgroup), gender (not included in gender subgroup), education level, BMI, marital status, smoking, drinking, DM, asthma, TC, and LDL.

**Fig. 3:**
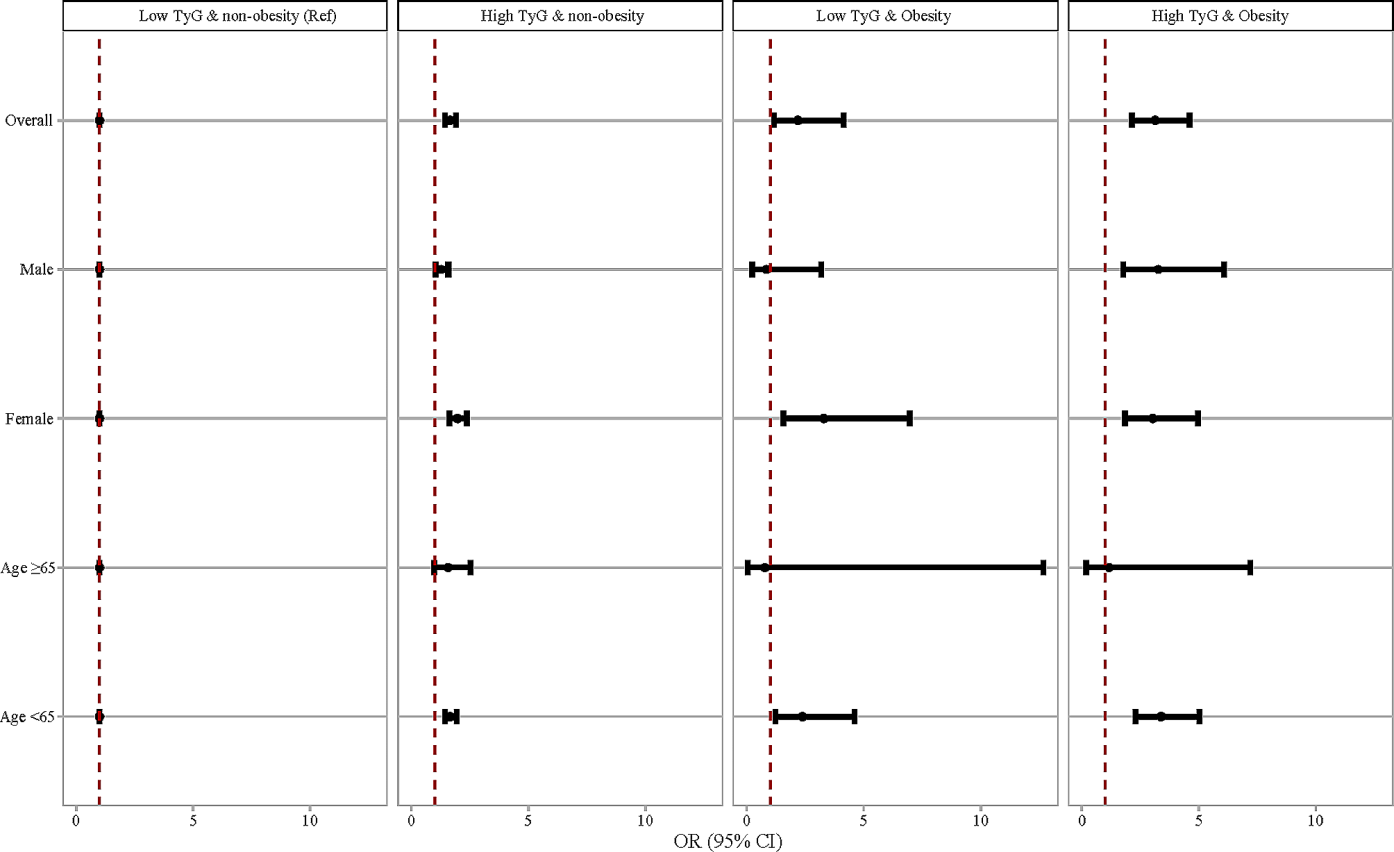
Interaction effect between TyG index and obesity on hypertension

Additionally, Fig. 3 showed the potential interaction effect between TyG index and obesity (recognized as BMI ≥ 30 kg/m^2^) on hypertension, and that among age and gender subgroups. We found that compared with low TyG index combined with non-obesity, high TyG index combined with non-obesity or high TyG index combined with obesity were both associated with higher odds of hypertension in the total study population, female subgroup, and age < 65 years old subgroup (all *P* < 0.05). The result indicated that there was a potential additive interaction between TyG index and obesity on hypertension.

## Discussion

This study based on the CHNS database and investigated the association between TyG index and hypertension among general population. The results showed that participants with higher TyG index level seemed to have higher odds of hypertension compared with those with low TyG index. This relationship was also found in persons who were female or aged < 65 years old. In addition, there was a potential additive interaction effect between TyG index and obesity on hypertension.

TyG index, an emerging measurable substitution of IR, is significantly and independently associated with prehypertension and hypertension after various confounding factors adjustment [11, 20]. In a 9-year longitudinal population-based study, the TyG index has been reported to be a good indicator for incident hypertension, and a higher TyG index was associated with an increased risk of subsequent incident hypertension [21]. Jian et al. [22] also confirmed an independent positive relationship between the TyG index and incident hypertension. Our findings indicated that adults who have a higher TyG index (> 8.41) seemed to have higher odds of hypertension compared with those have low TyG indexes. Differ from the previous studies, our study was a retrospective cohort study among the general population in China, and the participants were aged ≥ 18 years old while Jian’s study focus on persons at middle-age and older. We hope this finding could provide further evidence for the relationship between TyG index and hypertension and information for exploration of hypertension prevention in general population.

IR is the central link of metabolic diseases as well as a risk factor for cardiovascular disease (CVD) including hypertension. The mechanisms of the role of IR in hypertension are multifarious. IR is characterized of low-degree systematic inflammation, which may cause endothelial dysfunction, one of the initial pathogenic processes underlying arterial hypertension [23]. IR may also enhance salt absorption in the proximal tubule and cause water-sodium retention indirectly, resulting in hypertension [24]. In addition to the decline in efficiency of insulin to promote glucose uptake and utilization, metabolic disorders of lipid are also an important manifestation of IR, and adipose tissue may compensate for hyperinsulinemia and result in hypertension [25]. A cross-sectional study in Mexico indicated that the TyG index had higher sensitivity (96.5%) and specificity (85.0%) for detection of IR compared with the hyper insulinemic-euglycemic clamp test [26]. In fact, compared with the hyper insulinemic-euglycemic clamp test, TyG index could be easily obtained because it depends on inexpensive and routine measurement of the triglyceride and glucose. Mendia et al. [27] compared the ability of estimating IR between TyG index and HOMA-IR in healthy participants, and found that TyG index performed better with a sensitivity of 84.0% and a specificity of 45.0%. Wang et al. [28] also indicated that TyG index had a predictive performance on hypertension better than the TG/HDL-C ratio. Due to the superiority of TyG index as well as the simple and easy measurement of TG and FPG, the application of mass screening for TyG index in normotensive people is significant to the primary prevention of hypertension. However, the potential pathophysiological mechanisms of the association between TyG index and the occurrence of hypertension are still needed further investigation.

The results of subgroup analysis showed that the relationship between high TyG index and high odds of hypertension was also found in adults who were female or aged < 65 years old. Oppositely, Zhu et al. [20] given the opinion that people with older age (≥ 65 years old) had higher risk of hypertension when the TyG index was at a high level. A possible explanation for the age difference may be the hypertensive sub-types are age dependency and the prevalence of abnormal fat and glucose metabolism increases with age [29, 30]. The specific mechanism of the positive association between TyG index and hypertension is needed further exploration. Differ from our findings, Lee et al. [31] conducted a cross-sectional study in healthy Korean adults, and considered the TyG index was independently associated with increased arterial stiffness, the OR was 2.92 in men and 1.84 in women. Another study also found men showed larger regression coefficients and ORs of the TyG index in relation with increased arterial stiffness than women [32]. Generally, compared with women, men have more risk factors related to metabolic diseases. Such as, men were more likely to be smokers and drinkers, and had higher WC, serum uric acid, serum homocysteine, and lower eGFR. Significant interaction between TyG index and smoking on hypertension risk in males has been observed [33]. Experimental studies suggested that smoking may cause and aggravate IR mainly through stimulating the release of catecholamines and other anti-hormones, impairing the pathway of intracellular glucose metabolism, causing lipid metabolism disorders, and increasing vascular endothelial dysfunction, and finally result in the hypertension [34, 35]. However, the possible explanation for the gender inconsistency between our results and that of previous study may be the sex susceptibility in some specific contributing factors to IR, which is needed further clarification.

In addition, we explored the interaction between TyG index and obesity on hypertension, and the results showed that compared woth low TyG index combined with non-obesity, high TyG index combined with non-obesity/obesity was associated with higher odds of hypertension both in total study population and in female subgroup and aged < 65 years old subgroup. These findings indicated that there may be a potential additive interaction between TyG index and obesity on the risk of hypertension among general population. Similarly, Jian et al. [22] performed a cross-sectional study in Bengbu, China demonstrated an interaction between TyG index and abdominal obesity on hypertension risk. Another cross-sectional study in Henan, China also found interactions of TyG index and waist-to-height ratio and percent body fat on hypertension risk [28]. Compared with Jian’s and Wang’s study, our study expanded the regions of study population, and used a cohort study design. Obesity and IR share the common pathophysiological mechanisms in the development of hypertension. Obesity results in a milieu of pro-inflammatory and prooxidative, and promotes IR. Increased adipose tissue affects the regulation of blood pressure and the additional autocrine and paracrine activities of adipose tissue also contribute to inappropriate activation of the renin-angiotensin-aldosterone system and the sympathetic nervous system that promote microvascular remodeling, stiffness, a sodium retention that promote hypertension [36, 37]. Herein, the mechanisms of interaction between obesity and TyG index may because adipose tissue may compensate for hyperinsulinemia resulting in hypertension.

The relationship between TyG index and hypertension in general population was discussed in the current study, which further indicated that TyG index may be a potential early indicator to identify the odds of hypertension in clinical practice. Our study was based on the CHNS survey, which was conducted by a trained and certified team using standardized questionnaires which improved the credibility of the study data. However, there are some limitations in our study. This was a retrospective study, and some of the data collection used the questionnaires, which could not avoid the information bias. Although we tried our best to include as much as possible covariates, because of the missing of data on variables in the database, such as physical activity and menopause, the results could still influencing by the bias from other confounding factors. Also, due to the limitation of the database, some medication or dietary status such as lipid-lowering or hypoglycemic drugs and the measurement of hypertension related sodium intake could not take into consideration. Moreover, the study participants were only Chinese so that our findings may mainly reflect the association between TyG index and hypertension among Chinese population, which limited its general applicability for other races and regions. Therefore, it is necessary to conduct prospective studies in different races and regions to clarify the causal relationship between TyG index and the occurrence of hypertension, and further help to relieve the economic pressure, and improve the management and treatment implementation of hypertension in clinical.

## Conclusion

High TyG index was associated with high odds of hypertension among general population, and this relationship was also found in adults who were female or aged < 65 years old. Whether the TyG index can be used for early identification and high-risk population’s management of hypertension in clinical is needed further clarify.

### Electronic supplementary material

Below is the link to the electronic supplementary material.

Supplementary Material 1

## Data Availability

The datasets generated and/or analyzed during the current study are available in the CHNS database, https://www.cpc.unc.edu/projects/china/.

## Abbreviations

CVD: Cardiovascular disease
IR: Insulin resistance
CHNS: China Health and Nutrition Survey
CCDC: Chinese Center for Disease Control and Prevention
UNC: University of North Carolina
NINH: National Institute for Nutrition and Health
CPC: Carolina Population Center
BP: Blood pressure
TG: Triglyceride
FBG: Fasting blood glucose
ACC/AHA: American College of Cardiology and American Heart Association
SBP: Systolic blood pressure
DBP: Diastolic blood pressure
BMI: Body mass index
HDL: High density lipoprotein
LDL: Low-density lipoprotein
RCS: Restricted cubic spline
ORs: Odds ratios
CIs: Confidence intervals
