# Supplementary material for: Association between triglyceride-glucose index and hypertension: a cohort study based on the China Health and Nutrition Survey (2009–2015)
Source: BMC Cardiovasc Disord. 2024 Mar 19;24:168. doi: 10.1186/s12872-024-03747-9 (PMC10949779; doi:10.1186/s12872-024-03747-9)
Supplement: Supplementary file 1 — Supplementary Material 1 [file 12872_2024_3747_MOESM1_ESM.docx]

**Table S1. Description of missing variables**

| Variables | n (%) |
| --- | --- |
| Smoking status | 1 (0.03) |
| DM | 5 (0.14) |
| Asthma | 3 (0.09) |
| Weight, kg | 92 (2.61) |
| Height, m | 79 (2.24) |
| LDL, mg | 1 (0.03) |
| BMI, kg/m^2^ | 94 (2.67) |

DM: diabetes mellitus, LDL: low Density lipoprotein, BMI: body mass index

**Table S2. Sensitivity analysis of characteristics of participants before and after deletion of missing variables**

| Variables | After deletion  (n=3413) | Before deletion  (n=3521) | Statistics | *P* |
| --- | --- | --- | --- | --- |
| Age, years, Mean±SD | 47.89 ± 13.90 | 47.92 ± 13.92 | t=-0.11 | 0.914 |
| Gender, n (%) |  |  | χ^2^=0.032 | 0.859 |
| Male | 2388 (45.40) | 2454 (45.57) |  |  |
| Female | 2872 (54.60) | 2931 (54.43) |  |  |
| Education level, n (%) |  |  | χ^2^=0.002 | 0.963 |
| Lower middle school degree | 3952 (75.13) | 4048 (75.17) |  |  |
| Upper middle school degree | 1308 (24.87) | 1337 (24.83) |  |  |
| Marital status, n (%) |  |  | χ^2^=0.052 | 0.820 |
| Married | 1595 (30.32) | 1622 (30.12) |  |  |
| Others (divorced, separated, single, unknown) | 3665 (69.68) | 3763 (69.88) |  |  |
| Smoking, n (%) |  |  | χ^2^=0.000 | 0.994 |
| No | 3662 (69.62) | 3748 (69.63) |  |  |
| Yes | 1598 (30.38) | 1635 (30.37) |  |  |
| Drinking, n (%) |  |  |  | 0.926 |
| No | 2373 (69.53) | 2440 (69.30) |  |  |
| Yes | 1040 (30.47) | 1080 (30.67) |  |  |
| Unknown | 0 (0.00) | 1 (0.03) |  |  |
| DM, n (%) |  |  | χ^2^=0.021 | 0.884 |
| No | 5173 (98.35) | 5290 (98.38) |  |  |
| Yes | 87 (1.65) | 87 (1.62) |  |  |
| Asthma, n (%) |  |  | χ^2^=0.008 | 0.930 |
| No | 5211 (99.07) | 5329 (99.05) |  |  |
| Yes | 49 (0.93) | 51 (0.95) |  |  |
| Weight, kg, Mean±SD | 59.64 ± 10.70 | 59.63 ± 10.70 | t=0.04 | 0.968 |
| Height, m, Mean±SD | 161.02 ± 8.41 | 161.02 ± 8.41 | t=-0.01 | 0.989 |
| BMI, kg/m, Mean±SD | 22.92 ± 3.24 | 22.92 ± 3.24 | t=0.07 | 0.945 |
| TC, mg/dL, Mean±SD | 185.56 ± 37.90 | 185.56 ± 37.80 | t=0.00 | 0.997 |
| HDL, mg, Mean±SD | 56.01 ± 16.87 | 56.10 ± 17.50 | t=-0.29 | 0.772 |
| LDL, mg, M (Q_1_, Q_3_) | 113.47 ± 37.11 | 113.53 ± 37.31 | t=-0.07 | 0.943 |
| Insulin injection use, M (Q_1_, Q_3_) | 0.00 (0.00, 1.00) | 0.00 (0.00, 1.00) | Z=0.000 | 1.000 |
| Fasting glucose, mg/dL, Mean±SD | 94.52 ± 24.05 | 94.49 ± 24.02 | t=0.06 | 0.954 |
| Triglyceride, mg/dL, M (Q_1_, Q_3_) | 105.40 (71.74, 162.98) | 105.40 (71.74, 162.09) | Z=0.104 | 0.917 |
| TyG index, Mean±SD | 8.55 ± 0.68 | 8.55 ± 0.68 | t=0.09 | 0.932 |
| TyG index level, n (%) |  |  | χ^2^=0.028 | 0.866 |
| ≤8.41 | 2829 (53.78) | 2905 (53.95) |  |  |
| >8.41 | 2431 (46.22) | 2480 (46.05) |  |  |

SD: standard difference, DM: diabetes mellitus, TC: total cholesterol, M: median, BMI: body mass index, HDL: high density lipoprotein, LDL: low density lipoprotein, TyG index: triglycerides-glucose index

t: t-test, Z: rank sum test, χ2: chi-square test.
